# Suicide during Pregnancy with Rupture of the Uterus

**Published:** 1890-11

**Authors:** 


SUICIDE DURING PREGNANCY WITH RUPTURE OF THE UTERUS.—Neugebauer (*Centralblatt fur Gynakologie* p. 88, 1890) reports the case of a primipara nearly at term who committed suicide by throwing herself from the third story of a house upon the stone pavement. On examination, fracture of the skull and lacerated wounds of the head and face were found. The uterus could be outlined, but no symptom of labor was present. The patient perished in shock, and post mortem examination revealed rupture of the uterus, the child lying among the intestines covered by the mesentery. It had evidently occupied the position of breech-presentation during the life of the mother. The patient survived for several hours after her fall, during which time no hemorrhage could be discovered. The membranes remained unruptured, and probably occasioned the failure to diagnose rupture of the uterus. Although the mother sustained fracture of the skull, she twice sprang from the bed, and became partially conscious on one occasion. A detailed account of the injuries to the mother's pelvis accompanies the report of the case.

